# Involvement of the* Nucleus Incertus* and Relaxin-3/RXFP3 Signaling System in Explicit and Implicit Memory

**DOI:** 10.3389/fnana.2021.637922

**Published:** 2021-03-18

**Authors:** Isis Gil-Miravet, Aroa Mañas-Ojeda, Francisco Ros-Bernal, Esther Castillo-Gómez, Hector Albert-Gascó, Andrew L. Gundlach, Francisco E. Olucha-Bordonau

**Affiliations:** ^1^Unitat Predepartamental de Medicina, Facultat de Ciències de la Salut, Universitat Jaume I, Castelló de la Plana, Spain; ^2^Centro de Investigación Biomédica en Red de Salud Mental, CIBERSAM, Madrid, Spain; ^3^Department of Clinical Neurosciences, UK Dementia Research Institute, University of Cambridge, Cambridge, United Kingdom; ^4^The Florey Institute for Neuroscience and Mental Health, The University of Melbourne, Parkville, VIC, Australia

**Keywords:** neuropeptide, RXFP3, GABA, brainstem, amygdala, hippocampus

## Abstract

Telencephalic cognitive and emotional circuits/functions are strongly modulated by subcortical inputs. The main focus of past research on the nature of this modulation has been on the widespread monoamine projections to the telencephalon. However, the *nucleus incertus* (NI) of the pontine tegmentum provides a strong GABAergic and peptidergic innervation of the hippocampus, basal forebrain, amygdala, prefrontal cortex, and related regions; and represents a parallel source of ascending modulation of cognitive and emotional domains. NI GABAergic neurons express multiple peptides, including neuromedin-B, cholecystokinin, and relaxin-3, and receptors for stress and arousal transmitters, including corticotrophin-releasing factor and orexins/hypocretins. A functional relationship exists between NI neurons and their associated peptides, relaxin-3 and neuromedin-B, and hippocampal theta rhythm, which in turn, has a key role in the acquisition and extinction of declarative and emotional memories. Furthermore, RXFP3, the cognate receptor for relaxin-3, is a G_i/o_ protein-coupled receptor, and its activation inhibits the cellular accumulation of cAMP and induces phosphorylation of ERK, processes associated with memory formation in the hippocampus and amygdala. Therefore, this review summarizes the role of NI transmitter systems in relaying stress- and arousal-related signals to the higher neural circuits and processes associated with memory formation and retrieval.

## Introduction

Forebrain function is driven by subcortical ascending connections resulting in arousal activation allowing cognitive and emotional processes. The Ascending Reticular Activating Systems (ARAS) concept was postulated on the basis of the classical work of Moruzzi and Magoun (1949). In this pioneering research, upper brainstem stimulation resulted in the shift from high voltage synchronized sleep EEG to low voltage desynchronized EEG of wakefulness. Ascending pathways from the pontine-mesencephalic reticular projections to the thalamus were initially proposed as the central core of the ARAS (Steriade and Glenn, 1982). However, the complexity of the ascending projections arising from the ponto-mesencephalic reticular formation and the contribution of non-reticular brainstem structures lead to the consideration of the relevance of the basal forebrain as a key relay for these ascending systems in arousal performance (Parvizi and Damasio, 2001). In this context, the basal forebrain, including the medial septum (MS), was seen as a key element in driving hippocampal theta rhythm, a synchronizing hippocampal wave associated with movement (Vanderwolf, 1969).

The MS plays a central role in driving hippocampal theta rhythm as the endpoint of ascending projections arising from the mammillary bodies, median raphe, and (*reticularis pontis oralis RPO*; Bland et al., 1994; Vertes and Kocsis, 1997). All components of this ascending system are targeted by the pontine *nucleus incertus* (NI; Goto et al., 2001; Olucha-Bordonau et al., 2003), which led to the proposal that the NI was also a key element in driving hippocampal theta, and thus was a component of the ARAS (Olucha-Bordonau et al., 2003). At the same time, it was discovered that the rat NI contained neurons producing the neuropeptide, relaxin-3 (Burazin et al., 2002; Tanaka et al., 2005; Ma et al., 2007), which interacted with multiple stress and arousal transmitter systems (Ma et al., 2013, 2017b; Blasiak et al., 2015). Thus, the NI is a complex structure composed predominantly of GABAergic neurons in the rat (Ford et al., 1995; Olucha-Bordonau et al., 2003; Ma et al., 2007) and mouse (Smith et al., 2010) brain, and populations of NI GABAergic neurons co-express neuromedin-B (NMB; Lu et al., 2020; Nasirova et al., 2020), cholecystokinin (Kubota et al., 1983; Olucha-Bordonau et al., 2003) and/or neurotensin (Jennes et al., 1982), as well as relaxin-3, in both species. This neurochemical heterogeneity is also reflected physiologically, as a majority of relaxin-3 neurons in the rat NI increase their firing rate in response to CRF, while non-relaxin-3 neurons can increase or decrease their firing rate (Ma et al., 2013). Moreover, most relaxin-3 NI neurons preferentially fire at the initial ascending phase of the hippocampal theta rhythm, while non-relaxin-3 neurons do not display any relationship with hippocampal theta rhythm (Ma et al., 2013).

Concerning arousal modulation during wake/sleep function, it was widely proposed that ARAS promoting centers in the brainstem act *via* slow mechanisms driven by monoaminergic, cholinergic, and peptidergic systems (Jones and Webster, 1988; Burlet et al., 2002; Berridge and Waterhouse, 2003; Xu et al., 2004). This view was challenged by several experiments in which multiple ablations of these systems produced little effect on wake/sleep proportions. This resulted in a focus on the possibility that fast glutamatergic/GABAergic projections drive a more accurate control on arousal centers (Saper and Fuller, 2017). In this respect, the NI provides an ascending projection that contains the fast transmitter, GABA (Ford et al., 1995; Olucha-Bordonau et al., 2003), and several slower-acting peptidergic modulators (Ma et al., 2007; Ma and Gundlach, 2015, 2017b).

Notably, NI neurons project to most components of the ARAS, including those responsible for the subcortical triggering of hippocampal theta rhythm (Vertes and Kocsis, 1997; Tanaka et al., 2005; Ma et al., 2007; Smith et al., 2010), and thus are well-positioned to play a role in modulating memory acquisition and retrieval. In line with the widespread projections of the NI system, several lines of research have indicated its involvement in a range of functions, including stress (Banerjee et al., 2010; Ryan et al., 2013a), food intake (Ganella et al., 2013a; Lenglos et al., 2013; Calvez et al., 2017), alcohol addiction (Ryan et al., 2013b, 2014), arousal (Ryan et al., 2011; Ma et al., 2017a), and memory generation and retrieval (Nategh et al., 2015; Haidar et al., 2017, 2019; Szönyi et al., 2019; Lu et al., 2020).

Therefore, previous reviews of the neurobiology of the NI and its relaxin-3 signaling system have focused on a likely role in stress and arousal (Ryan et al., 2011; Ma and Gundlach, 2015), feeding behavior (Ganella et al., 2013b), and a possible involvement in mental illnesses (Smith et al., 2014; Kumar et al., 2017) with a summary of associated relaxin-3 signaling *via* RXFP3 (Ma et al., 2017b; Olucha-Bordonau et al., 2018). In this review, our goal was to discuss the basis on which these different functions are linked to memory processes and how relaxin-3/RXFP3 signaling supports such functions. For example, memory is relevant when searching for places containing food resources. However, while there is a perception that brainstem nuclei, including the NI, provide general widespread modulation of telencephalic circuits/functions, this idea has been challenged by the recent demonstration of a likely role of NI GABAergic neurons in selecting relevant memory information (Szönyi et al., 2019). Therefore, here we review the main findings related to the role of the NI and the relaxin-3/RXFP3 signaling system in explicit and implicit memories, and discuss current gaps in our knowledge, along with possible developments and future directions that will help to better determine the precise role of this system in learning processes.

## Nucleus Incertus/Relaxin-3 Innervation of Memory-Related Centers

Relaxin-3 was the last member of the relaxin family to be discovered (Bathgate et al., 2002; Burazin et al., 2002; Gundlach et al., 2013), but represents the ancestral form of the other relaxin family peptides. Similar to insulin peptides, mature relaxin-3 is composed of an A- and B-chain linked by two disulfide bonds. The B-chain peptide contains the characteristic “RXXXRXX(I/V)” sequence, which has been maintained in all vertebrates (Wilkinson and Bathgate, 2007). Two years after the peptide discovery, the G_i/o_ protein-coupled receptor, GPCR135, was identified as the cognate receptor for relaxin-3 and was renamed as the relaxin family peptide 3 receptor (RXFP3). Relaxin-3 mRNA expression was found to be most abundant in the brain (Burazin et al., 2002), with some expression in other organs such as the lungs, liver, spleen, thymus, and gonads (Bathgate et al., 2002).

The relaxin-3 expression is predominantly located in neurons of the NI of the pontine tegmentum in rats, mice, and macaque (Burazin et al., 2002; Ma et al., 2009b; Smith et al., 2010). Also, studies of peptide mRNA and immunoreactivity in rat and mouse revealed three additional relaxin-3-positive neural groups located in the lateral part of the *substantia nigra*, the ventrolateral part of the periaqueductal gray (PAG), and the pontine raphe nucleus (Tanaka et al., 2005; Ma et al., 2007; Smith et al., 2010). Notably, while in the rat brain, most (~50%) relaxin-3 neurons are concentrated in the NI over a rostrocaudal extension of 750 μm; in the PAG, these neurons appear dispersed along a rostrocaudal column extending approximately 3,000 μm. This column is ventrolaterally located to the aqueduct, but in 20–40 μm coronal sections, only 3–4 relaxin-3 positive neurons appear in each hemisphere, but the total number of relaxin-3 neurons represents 50% of the number in the NI. Thus, these neurons should be considered as a relevant source of relaxin-3 in the brain (Blasiak et al., 2013), particularly as part of the brainstem intrinsic circuitry (Nasirova et al., 2020). Furthermore, anatomical data suggest that the pontine raphe nucleus is a rostral extension of the NI (Nasirova et al., 2020). While it is often considered a serotoninergic nucleus (Hale and Lowry, 2011), the number of neurons positive for serotonin (5-HT) in the pontine raphe nucleus is lower than the number of relaxin-3 positive neurons (Tanaka et al., 2005). In contrast, the number of relaxin-3 neurons in the lateral part of the *substantia nigra* is very low, even in colchicine-treated rats, with only 2–3 neurons observed in consecutive 40 μm sections (Tanaka et al., 2005; Smith et al., 2010; Blasiak et al., 2013). Thus, apart from an earlier study of the PAG to intergeniculate nucleus relaxin-3 pathway and its likely role in circadian rhythms (Blasiak et al., 2013), the focus of research on relaxin-3 neurobiology has largely centered on NI relaxin-3 neurons.

NI has widespread connections to telencephalic centers that play a major role in the acquisition, storage, and retrieval of explicit (hippocampal-dependent) and implicit (amygdala-dependent) memories. NI connections include subcortical centers that modulate hippocampal and amygdala function, including the MS, supramammillary nuclei, and both the median and dorsal raphe nuclei in the rat (Goto et al., 2001; Olucha-Bordonau et al., 2003), mouse (Smith et al., 2010; Szönyi et al., 2019; Lu et al., 2020; Nasirova et al., 2020) and non-human primate (Ma et al., 2009b).

As mentioned, and discussed in detail below, the NI contains heterogeneous populations of GABAergic neurons containing different neuropeptides, including relaxin-3, NMB, or cholecystokinin. Generally, the precise role of these peptides in NI function remains to be investigated, although multiple studies have focused on the involvement of relaxin-3 in several modalities.

## Relaxin-3/Rxfp3 Signaling and Interference with Plastic Memory Processes

While research over the last two decades has implicated the relaxin-3/RXFP3 system in a wide variety of functions, including modulation of stress, anxiety, foraging, and addictive and social behaviors (see Ma et al., 2017b; Olucha-Bordonau et al., 2018), for this review we examined the evidence for a specific role of NI transmitter systems, particularly relaxin-3/RXFP3, in relaying stress- and arousal-related signaling to higher neural centers associated with memory formation and retrieval.

ERK phosphorylation and cAMP synthesis/degradation are two processes directly related to plastic memory changes occurring in the hippocampus and the amygdala. The activation of protein kinase A (PKA) by cAMP has been associated with long-term potentiation (LTP) in the hippocampus (Frey et al., 1993; Huang et al., 1994; Abel et al., 1997) and is required for long-term spatial memory in the Morris water maze (MWM; Abel et al., 1997). Similarly, the cAMP/PKA signaling pathway is required and sufficient to induce LTP in the thalamo-amygdala and cortico-amygdala systems (Fourcaudot et al., 2008). Similarly, PKA is required for plastic changes associated with emotional memories in the amygdala associated with fear conditioning (Goosens et al., 2000; Moita et al., 2002). Thus, inhibition of cAMP levels by RXFP3 activation may impair the formation of memories under these or similar experimental and physiological conditions.

In the adult brain, ERK phosphorylation is also a process associated with memory generation, depending on the specific circuits involved. In the hippocampus, ERK phosphorylation has been found to mediate plastic changes induced by LTP in hippocampal slices (Selcher et al., 2001). Similarly, the formation of the hippocampal cognitive map is associated with ERK phosphorylation in specific hippocampal regions (Blum et al., 1999; Selcher et al., 1999). ERK phosphorylation also mediates LTP-related plasticity induced by thalamo-amygdala stimulation of the fear circuit (Apergis-Schoute et al., 2005). This process is also associated with fear memory acquisition and consolidation (Schafe et al., 2000). ERK2 mutant mice have been characterized as displaying impairment in social recognition and other social behavior problems (Satoh et al., 2011).

**Figure 1:**
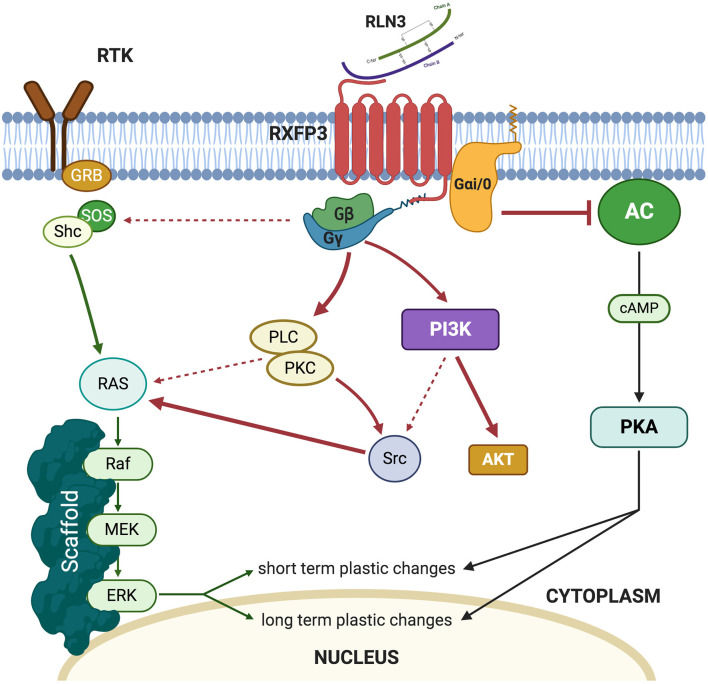
Summary of the intracellular pathways triggered by relaxin-3 (RLN3) activation of RXFP3.

Relaxin-3 binds to RXFP3 with high affinity (K_d_ 300 pM) and *in vitro* studies in Chinese hamster ovary (CHO)-K1 cells overexpressing RXFP3 revealed that the addition of recombinant relaxin-3 to the media resulted in inhibition of cAMP accumulation (Liu et al., 2003). In similar experimental models, it was demonstrated that RXFP3 activation resulted in the activation of the MAP-ERK pathway in a PKC-dependent manner (Van der Westhuizen et al., 2005, 2007; Figure 1). *In vivo*, intracerebroventricular (icv) infusions of an RXFP3 agonist increased ERK phosphorylation in the septum (Albert-Gascó et al., 2017) and amygdala (Albert-Gasco et al., 2019) reaching a peak at 20 min and returning to baseline (control) values by 90 min. However, this pERK activation was largely produced in RXFP3 mRNA negative neurons in these regions, suggesting this *in vivo* ERK activation was an indirect effect (Albert-Gasco et al., 2019).

Under normal conditions, pERK activation is known to be produced in the medial amygdala (MeA) during a social interaction test and social recognition memory without relaxin-3/RXFP3 modulation. This is a likely reason why 20 min after RXFP3 agonist infusion, the MeA still displays a considerable amount of pERK positive neurons, which do not express RXFP3. These pERK-positive neurons are likely to be a result of social behavior rather than an indirect effect of relaxin-3/RXFP3 system activation. This argument is supported by the fact that activating RXFP3 during this behavioral paradigm significantly increased the number of RXFP3-expressing neurons which contained pERK compared to controls, suggesting a direct effect on these neurons. However, all studies where RXFP3 agonists increased pERK were quantified after assessing retrieval of memories (Albert-Gascó et al., 2017; Albert-Gasco et al., 2019). So, it is possible, given the transient nature of MAPK activation, that these approaches are likely to have missed the complete direct increase of pERK in RXFP3-expressing neurons due to RXPF3 agonist activation. These results support the hypothesis that experimental timing is crucial to determine the role and adequately delimit the downstream pathways which mediate relaxin-3/RXFP3 induced neuronal plasticity. Another relevant aspect that remains to be assessed is how RXFP3 agonist activation modulates neuronal firing patterns within its different targeted areas. Electrophysiological studies in brain slices which focused on the hypothalamus and the *bed nucleus of the stria terminalis* demonstrated a reversible inhibition of neuronal firing (Kania et al., 2017, 2020; Ch’ng et al., 2019).

Thus, a balance between inhibition of cellular cAMP levels and ERK phosphorylation in particular neurons of the amygdala and hippocampus may result in particular aspects of experienced events being filtered out and others being enhanced in a particular environmental or social context. Currently, there is a lack of data on the effects of RXFP3 activation on the cAMP/PKA system or the electrophysiological consequences of RXFP3 activation in the amygdala and hippocampus, and more research is needed to decipher these key aspects of this dual cell signaling system and the nature of its complex regulation by RXFP3.

Furthermore, little is known about the effect(s) of the presumed co-release of GABA and relaxin-3. EM images of NI terminals are reported to contain the oval vesicles of GABAergic terminals and contain dense-core vesicles (DCV) associated with peptide storage (Tanaka et al., 2005; Ma et al., 2009a; Olucha-Bordonau et al., 2012). NI neurons including those that are relaxin-3-positive are GABAergic, and GABA and peptides may act in co-operation or in opposition at postsynaptic targets, depending upon the nature of the peptide and its receptor(s). Unfortunately, little is known about the specific dynamic modulation of relaxin-3 release from DCV at nerve terminals or the precise type of stimulation that leads to relaxin-3 release. More generally, it is thought that DCV are released after high-frequency stimulation (Hartmann et al., 2001), and if this is the case for GABA/relaxin-3-positive neurons, train stimulation of the NI would lead to the release of relaxin-3 at nerve terminals. In neuropeptide systems, the neuropeptide tends to be co-released with other neurotransmitters, being GABA in the case of relaxin-3 neurons. In most cases, transmitter and peptide release do not occur as separate events but can have different behavioral outcomes (Hartmann et al., 2001), and therefore it is likely that the NI/relaxin-3 system modulates GABAergic signaling, synergizing with its effects, given that RXFP3 is a G_i/o_-protein-coupled receptor. However, more experiments are needed to fully understand the dynamics of the NI/relaxin-3/ GABA system.

## Nucleus Incertus and Hippocampal Theta Rhythm

Early seminal studies demonstrated that when a rat enters and explores a new environment, a cognitive map is formed in the hippocampus, whereby groups of cells fire specifically when the rat passes along a particular place field (O’Keefe and Dostrovsky, 1971). Directly related to the emergence of place cells, locomotion during the active exploration of the context generates a synchronic wave of 4–12 Hz called theta rhythm (Vanderwolf, 1969; Buzsáki et al., 1983). A particular feature of the link between the cognitive map and hippocampal theta rhythm is the theta precession. When a rat approaches a particular place field, place neurons fire regularly following theta rhythmicity, but the spikes move progressively to an anterior phase of theta as the rat passes through the place field (O’Keefe and Recce, 1993). Thus, while an animal moves along a path, a set of place cells fire sequentially according to the phase of the local field potential, which is known as theta sequences (O’Keefe and Recce, 1993; Drieu and Zugaro, 2019). In contrast, theta precession and theta sequences have been observed in circumstances where locomotion does not necessarily involve a change in space, like wheel-running, treadmill-running, or virtual reality (Pastalkova et al., 2008; Royer et al., 2012; Chen et al., 2013). All these data suggest that theta precession and theta sequences are not only linked to space and context, but all components of episodic memory (Jaramillo and Kempter, 2017). Along with these processes, a cognitive map is formed, stored, and eventually retrieved in the hippocampus containing an egocentric and an allocentric component. The egocentric component is taken from reference to the self-movement, while the allocentric component is taken from external references (Burgess, 2006).

Theta can be triggered *via* different mechanisms. First, oscillation at theta frequencies can be elicited from membrane resonance provided by intrinsic properties of various voltage-dependent channels (Evstratova et al., 2011). Second, theta can arise from intrinsic hippocampal connections including rhythmic inputs from the entorhinal cortex targeting the dentate gyrus, CA3 or CA1 fields (Alonso and García-Austt, 1987a, b), activation of CA1 cells from CA3 inputs (Kamondi et al., 1998), and interneuronal networks (Goutagny et al., 2009). Finally, theta can be elicited from ascending subcortical projections arising mainly from the MS and the supramammillary nucleus, the median raphe, and the NI (Kocsis and Vertes, 1997; Vertes and Kocsis, 1997; Vertes et al., 1999; Nuñez et al., 2006; Tsanov, 2015; Ruan et al., 2017).

**Figure 2:**
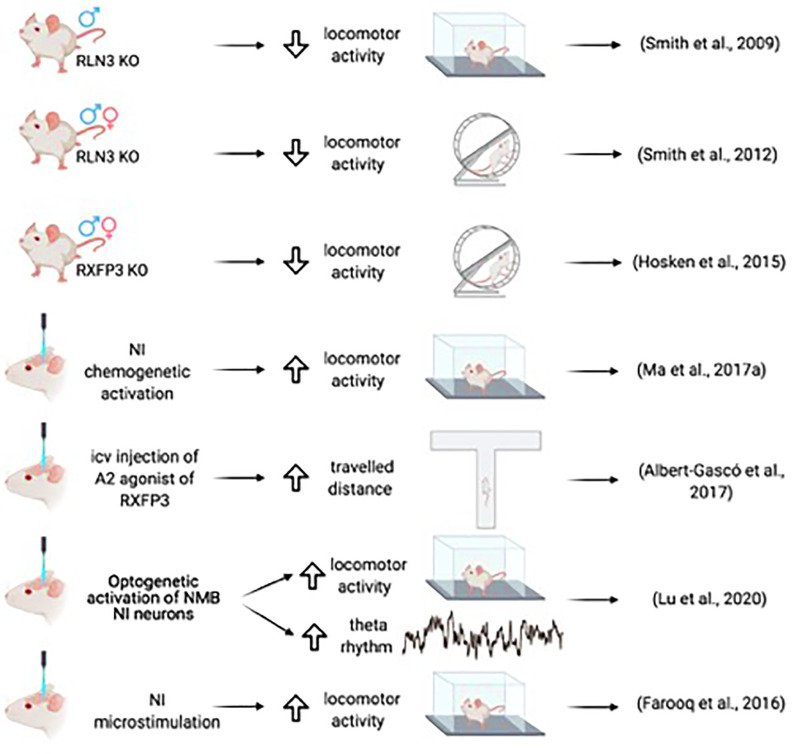
Summary of the main effects of NI-RLN3-RXFP3 activation on locomotor activity.

The role of the NI in theta rhythm generation was first hypothesized based on its anatomical connectivity (Olucha-Bordonau et al., 2003). Ascending connections arising from the NI target sequentially the median and dorsal raphe, supramammillary nucleus, MS, entorhinal cortex, and hippocampus, and each of these nodes provides additional ascending connections to the upstream components of the system, converging in the hippocampus (Olucha-Bordonau et al., 2003). In urethane anesthetized rats, electrical stimulation of the NI elicited an increase in the theta band of the local field potential and at the same time a decrease in the delta band. Moreover, lesions of the NI eliminated the theta rhythm induced by sensory stimulation or electrical stimulation of the *nucleus reticularis pontis oralis* (RPO; Nuñez et al., 2006). Similarly, infusion of R3/I5, a selective RXFP3 agonist (Liu et al., 2005), into the MS elicited hippocampal theta rhythm in urethane anesthetized rats; and this increase was prevented by a prior infusion of R3(BΔ23–27)R/I5, a selective RXFP3 antagonist (Kuei et al., 2007). Furthermore, infusion of the RXFP3 antagonist into the MS disrupted the increase in hippocampal theta produced by RPO stimulation (Ma et al., 2009a; Figure 2).

Indeed, the relationship between theta oscillations in the hippocampus and the NI has been consistently observed using acute recordings in urethane anesthetized rats. Strong coherence in the local field potential between the hippocampus and the NI was observed following sensory or RPO stimulation (Cervera-Ferri et al., 2011). Furthermore, it was observed that a specific type of electrophysiologically characterized neuron in the NI increased firing after sensory stimulation-induced hippocampal theta and was phase-locked with hippocampal theta (Martínez-Bellver et al., 2015). Chemically characterized neurons also displayed a differential firing in relation to the hippocampal theta. While relaxin-3 negative neurons of the NI did not fire in a particular way in relation to theta, relaxin-3 positive neurons fire mainly in the ascending phase of hippocampal theta (Ma et al., 2013). Indeed, a causal relationship was identified between the theta phases of the hippocampus and the NI when Granger causality analysis was applied, in which NI activity induced a reset of hippocampal theta (Martínez-Bellver et al., 2017). As an anatomical substrate of this electrophysiological coupling between the hippocampus and the NI, asymmetric bidirectional connections between these structures were identified in the rat, with a stronger ascending NI to hippocampus projection than the opposing descending connection (Sánchez-Pérez et al., 2015). In fact, in a more recent study in mice, specific optogenetic stimulation of medial septal projections to the NI induced an increase in theta band power (Lu et al., 2020).

The involvement of the NI in hippocampal theta rhythm has been further elucidated in two transgenic mouse models (Szönyi et al., 2019; Lu et al., 2020). In one case (Szönyi et al., 2019), the investigators stimulated overall NI neurons, and in the other case, the investigators took advantage of a distinct neurochemical composition of the NI population of neurons using optogenetics (Lu et al., 2020). Szönyi and colleagues targeted the broad population of NI GABAergic neurons in vGAT-Cre mice and observed that optogenetic stimulation of the NI induced a decrease in theta power recorded in the CA1 field that was associated with effects on local somatostatin interneurons (Szönyi et al., 2019). In contrast, Lu and colleagues produced a transgenic mouse expressing Cre recombinase under the control of the NMB promotor (Lu et al., 2020), as NMB is widely expressed in mouse NI neurons (Chronwall et al., 1985). Optogenetic stimulation of NI NMB neurons induced an increase in theta power recorded in the CA1 field and a concomitant increase of locomotor activity (Lu et al., 2020). The opposing effects of the NI stimulation on theta might be explained by the assessment of different neuronal targets. However, most neurons of the NI express glutamic acid dehydrogenase (GAD) in the rat (Olucha-Bordonau et al., 2003; Ma et al., 2007) and mouse (Szönyi et al., 2019), and a recent report confirms that all NMB neurons are vGAT-positive (i.e., GABAergic; Nasirova et al., 2020). However, the surrounding nuclei contain GABA neurons (Nasirova et al., 2020) that when stimulated, as occurs in the vGAT-Cre mouse (Szönyi et al., 2019) could produce complex interactions leading to different behaviors to those obtained from specific activation of NMB neurons (Lu et al., 2020).

## Nucleus Incertus/Relaxin-3 Induction of Locomotor Activity and Arousal

In awake animals, a major component of hippocampal theta derives from locomotor activity (Vanderwolf, 1969). There is a direct non-linear relationship between the speed of movement and theta rhythm so that at higher speeds a harmonic of 16 Hz appears along with a series of higher frequency harmonics (Sheremet et al., 2016). There is also evidence of a relationship between locomotor activity, behavioral arousal, and NI activity (Ryan et al., 2011; Farooq et al., 2016).

In terms of interactions with other central arousal systems, NI neurons in the rat express a high density of CRF receptor 1 (CRF1; Bittencourt and Sawchenko, 2000; Van Pett et al., 2000), consistent with the proposal that CRF1 activation in the NI represents a mechanism for distributing a CRF-elicited arousal response during stressful conditions (Tanaka et al., 2005). Indeed, specific stressors have been shown to increase NI activity (Calvez et al., 2016). Furthermore, NI neurons in the rat express the orexin receptors, OX1 and OX2 (Blasiak et al., 2015), which represents another signaling mechanism for delivering arousal-related stimuli to the NI. Orexin-A application induced depolarization of NI neurons expressing relaxin-3 (Blasiak et al., 2015). Interference with orexin signaling by injection of an OX2 antagonist (but not OX1 antagonist) into the NI impaired the reinstatement of alcohol-seeking induced by the anxiogenic drug, yohimbine, in alcohol-preferring rats. Application of the same OX2 antagonist prevented NI cell depolarization induced by orexin-A in an *ex vivo* slice preparation (Kastman et al., 2016). Thus, the NI could mediate the effect of orexins on food- and alcohol-seeking by enhancing locomotor activity and theta-related processes.

A relationship between the NI and the associated relaxin-3 system and arousal and locomotion was further implied by studies of relaxin-3 knockout (Rln3 KO) mice. Both male and female KO mice displayed a hypoactive phenotype on voluntary, home-cage running wheels during the dark phase (Smith et al., 2012), a finding in agreement with the subsequent observation that male and female Rxfp3 KO mice displayed hypoactivity in the dark phase (Hosken et al., 2015; Figure 2).

A direct relationship between arousal and locomotion was demonstrated in studies of microstimulation of the NI in conscious rats. Ipsilateral microstimulation of the NI at a high frequency increased speed, mobility, and rotations at short latencies that lasted for a short time after stimulus cessation (Farooq et al., 2016). Sustained chemogenetic activation of the NI in rats also produced increased locomotor activity in the home cage and a novel environment, and desynchronized cortical EEG corresponding to activation of arousal mechanisms. Furthermore, during the retrieval of fear memory, chemogenetic activation of NI neurons resulted in increased head-scan and vigilance behavior (Ma et al., 2017a). In mice, increased locomotor activity was observed following the optogenetic activation of NMB NI neurons, in parallel with increased hippocampal theta rhythm (Lu et al., 2020).

Thus, several findings using different approaches, in rats and mice, point to a direct association between locomotor activity and hippocampal theta rhythm and reveal that both can be triggered by the activation of NI neurons. Notably, although anatomical studies have focused on ascending connections, a dense NMB plexus occurs in the inferior olivary nucleus (Lu et al., 2020) that could mediate the interplay between theta and arousal locomotion and cerebellar processing. Furthermore, this plexus is relaxin-3 negative (Nasirova et al., 2020), which suggests that different populations of NI neurons could play different roles in the modulation of theta rhythm activity concerning locomotion.

## Nucleus Incertus in Hippocampal-Dependent Declarative Memory

Declarative memory is the capacity to recall information about past facts, places, and events, and primarily depends on the integrity of the hippocampus. Given that a central role of the NI and its associated peptidergic systems is the modulation of hippocampal theta rhythm, it is logical that the NI may be involved in the acquisition and retrieval of declarative and spatial memories. The clinical features studied in the famous HM patient reported a key role of the hippocampus in declarative memory (Squire, 2004), but the strong effect of location and space on hippocampal cell activity suggests that space is of primary importance to declarative memory formation and must be considered as the central element in episodic and semantic memories (Buzsáki and Moser, 2013).

A likely direct role for the NI in spatial memory processes was demonstrated by Nategh and colleagues in studies in which they produced a transient inactivation of the NI in rats by local lidocaine injection before the performance of different aspects of the Morris water maze (MWM) test (Nategh et al., 2015). Lidocaine inactivation of the NI impaired both the acquisition and retrieval of the memory of the location of the submerged platform, but not memory consolidation, assessed in the spatial reference memory test (Nategh et al., 2015). Also, intra-NI lidocaine disrupted working memory in the MWM learning protocol (Nategh et al., 2015). These studies did not determine the site(s) at which NI inactivation produced these effects, which potentially include the hippocampus and other neural nodes within the septohippocampal system such as the supramammillary nucleus, MS, or entorhinal cortex, although the poor performance in lidocaine-treated rats during acquisition and retrieval was shown to result in decreased activation of both c-*fos* and pCREB in the pyramidal layer of the CA1 hippocampal field (Nategh et al., 2015). However, transient inactivation with lidocaine does not provide a selective assessment of the participation of the NI in these processes, as surrounding areas may be also affected. Currently, studies using chemogenetic inactivation or activation of the NI on performance in the MWM have not been reported.

**Figure 3:**
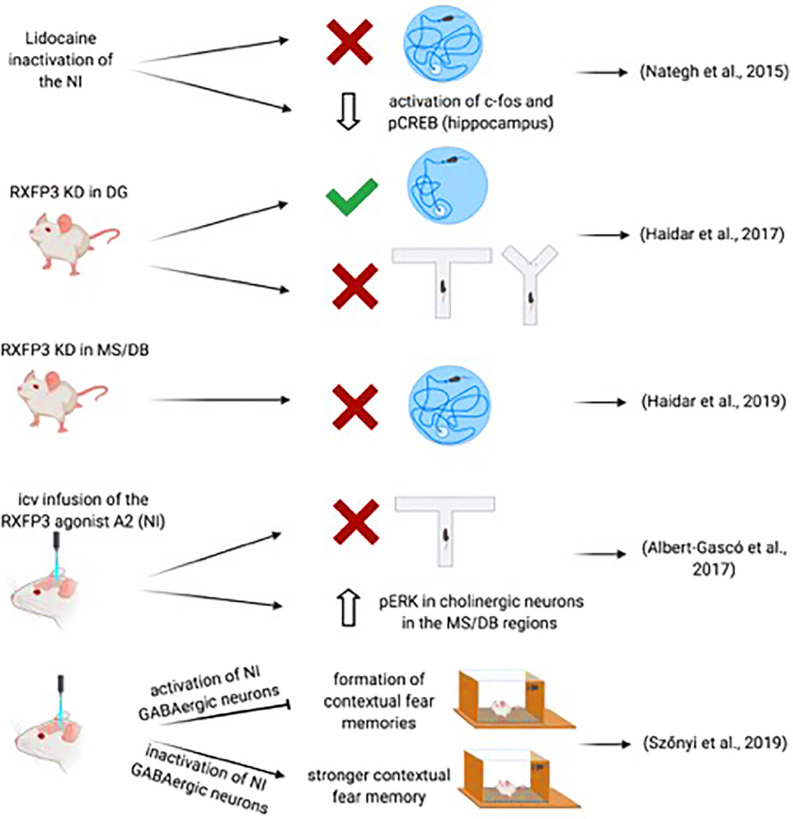
Summary of data demonstrating the involvement of the NI-RLN3-RXFP3 system in declarative and contextual memory.

However, possible involvement of NI-related, relaxin-3/RXFP3 signaling has been explored. Thus, while specific Cre-dependent depletion of RXFP3 from somatostatin interneurons in the dentate gyrus of mice expressing a floxed-Rxfp3 did not result in impairment of performance of the MWM memory test (Haidar et al., 2017); RXFP3 depletion in the MS of these mice resulted of impairment of memory formation and retrieval in the MWM (Haidar et al., 2019; Figure 3). More specifically, AAV-Cre induced deletion of RXFP3 in the dentate gyrus of the hippocampus in floxed-Rxfp3 mice produced an impairment in an appetitive T-maze task and spatial working memory in a continuous spontaneous alternation test in a Y-maze (Haidar et al., 2017), suggesting that the relaxin-3-containing inputs to somatostatin- and GABA-positive neurons in the hilar region could regulate hippocampal and cognitive processing associated with these tasks (Haidar et al., 2017). Similarly, depletion of RXFP3 in the medial septum/diagonal band of Broca (MS/DB) in the floxed-Rxfp3 mice produced impairment of the long-term reference memory in the MWM (Haidar et al., 2019). Consistent with these studies, icv infusion of the RXFP3 agonist, RXFP3-A2 resulted in impairment of spatial working memory in rats in a delayed spontaneous alternation T-maze test (Albert-Gascó et al., 2017). Additionally, RXFP3-A2 administration increased pERK immunostaining in cholinergic (ChAT-positive) neurons in the MS/DB regions (Albert-Gascó et al., 2017). Together these and other data indicate that RXFP3 signaling can regulate cognitive processing activity in the hippocampus and MS/DB *via* NI relaxin-3 projections.

Further support for the involvement of the NI in the regulation of memory-related hippocampal and septal circuits has been obtained in recent studies employing optogenetics and transgenic mouse lines. The study of Szönyi et al. (2019) reported that optogenetic activation of NI GABAergic neurons impaired the capacity for creating contextual associations to an unconditioned stimulus (US) *via* inhibition of hippocampal somatostatin interneurons. Furthermore, while a synchronized activation of NI GABAergic neurons generated an inhibition of the formation of contextual fear memories, inhibition of NI GABAergic neurons produced a stronger contextual fear memory formation; this effect could be enhanced by concurrent inhibition of excitatory MS neurons by GABAergic NI collaterals (Szönyi et al., 2019). However, this study used a GAD-Cre mouse in which NI activation-inactivation is produced after the infusion of an AAV-floxed-channelrhodopsin II (ChRII) into the NI. Thus, this manipulation may, in fact, affect other GABAergic neurons in the area. In this experimental approach, the activation could also be extended to intrinsic non-projecting GABA neurons and GABA neurons in other nuclei in the region directly or indirectly projecting to forebrain areas involved in memory processes (Wirtshafter and Stratford, 1993). In a more recent study, activation of NMB-positive, GABAergic neurons in the NI of mice increased locomotor activity, arousal, and hippocampal theta rhythm (Lu et al., 2020). Lu and colleagues observed that the NI-induced effects within the MS were quite specific, as activation of MS glutamatergic and cholinergic neurons increased locomotor speed, arousal, and theta power, whereas activation of MS GABAergic did not induce locomotion (Lu et al., 2020).

Although it appears clear there is a direct involvement of the NI in memory processes, as revealed by its impact on hippocampal theta rhythm and contextual perception and memory, there remain gaps in our knowledge related to the exact role of this intriguing signaling system. Most research so far has centered on the septum and hippocampus. However, other targets of the NI including the supramammillary nucleus, the median raphe, and the interpeduncular nuclei also provide ascending innervation to the septohippocampal system, thus modulating or actively driving, theta and memory systems. Similarly, the effect of the NI on these intermediary targets has not been explored. For example, the supramammillary nucleus may play a pivotal role, as it is directly involved in modulating the frequency of hippocampal theta rhythm (Kocsis and Vertes, 1994, 1997; McNaughton et al., 1995; Pan and McNaughton, 1997). Furthermore, the supramammillary nucleus has been reported to be involved in foraging mechanisms in response to ghrelin signaling (Le May et al., 2019) and RXFP3 is expressed in this region. Also, the supramammillary nucleus is involved in processing contextual and social novelty signals conveying it to the CA2 and dentate gyrus of the hippocampus, respectively (Chen et al., 2020), spatial memory retrieval (Li et al., 2020), and theta and gamma oscillations during REM sleep (Billwiller et al., 2020). Thus, the supramammillary nucleus, like the NI, is implicated in spatial and memory processing, and in activating food-seeking circuits. Notably, however, the NI is a heterogeneous nucleus containing different kinds of neurons. In fact, in a transgenic mouse expressing Cre recombinase under the control of the relaxin-3 promotor, a co-injection of a GFP-Cre-On and a TdT-Cre-Off viral vector, it was possible to differentiate the relaxin-3 positive from the relaxin-3 negative projections, and there is some mismatch between these types of fibers in the supramammillary and the interpeduncular nuclei (Nasirova et al., 2020). Thus, further studies are needed to discriminate between the specific roles of each neuronal type in the NI in particular aspects of memory formation consolidation and/or retrieval in various experimental species.

## Nucleus Incertus in Amygdala-Dependent Implicit Memories

Innervation of the medial and extended amygdala (MeA) by the NI in the rat is consistent with a role for this ascending system in emotional processing. The distribution of anterogradely-labeled nerve fibers following tracer injections into the NI and the presence of relaxin-3 fibers revealed a high level of labeling in the medial and extended amygdala, where they make contact with calcium-binding protein-positive neurons (Santos et al., 2016). By contrast, the basolateral and cortical amygdala are essentially devoid of NI and relaxin-3 fibers (Santos et al., 2016). Notably, the central amygdala and the lateral divisions of the *bed nucleus of the stria terminalis* essentially lack fibers, whereas *in situ* hybridization studies identify a high density of RXFP3 mRNA-positive neurons in both the central amygdala and/or the lateral nuclei of the *bed nucleus of the stria terminalis* in rat and mouse (Ma et al., 2007; Smith et al., 2010). However, a comparison of the density and distribution of relaxin-3 fibers in rats and mice reveal that while there is a similar distribution, the density of fibers is lower in mice than in rats (Ma et al., 2007; Smith et al., 2010). Additionally, new studies in NMB-Cre (Lu et al., 2020) and relaxin-3-Cre (Nasirova et al., 2020) mice did not describe the presence of NI fibers in the amygdala area.

**Figure 4:**
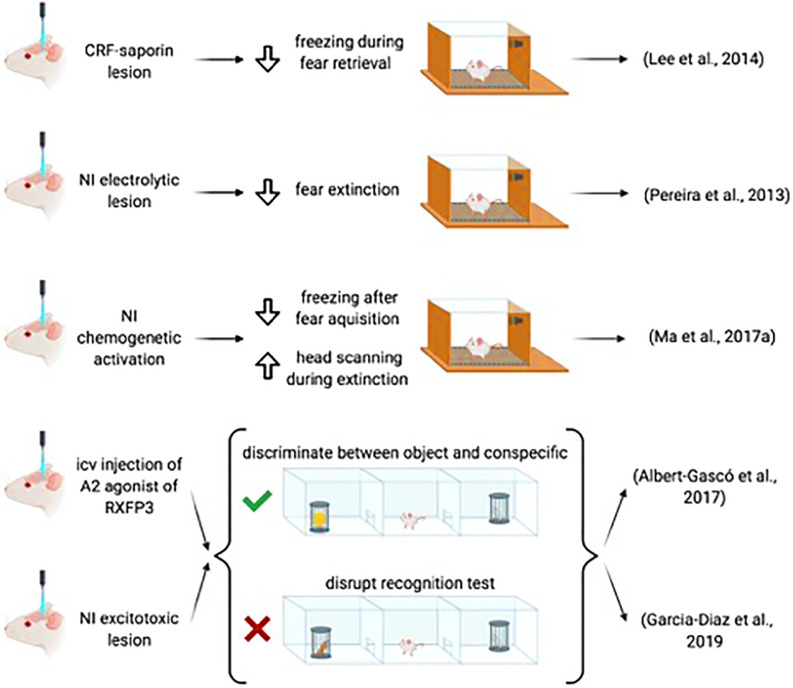
Summary of the data demonstrating the involvement of the NI-RLN3-RXFP3 system in social and emotional memory.

The occurrence of NI/relaxin-3 fibers and RXFP3 in the amygdala suggested a possible effect of this system on fear conditioning processes, which has been investigated in some initial studies. In rats in which CRF1-expressing neurons of the NI were lesioned using CRF-saporin, acquisition of a fear response to an auditory tone was not altered, but during retrieval, lesioned rats displayed longer freezing than sham rats (Lee et al., 2014). Similarly, electrolytic lesions of the NI did not alter fear acquisition but impaired fear extinction (Pereira et al., 2013). Furthermore, NI activation by CNO administration in rats injected previously with an AAV1/2-hM3Dq into the NI resulted in a significant reduction of freezing after the fear acquisition. However, this reduction of the fear response was accompanied by an increase in head scanning during extinction tones (Ma et al., 2017a; Figure 4). Notably, concerning the latter effect, CNO may have also activated surrounding pontine centers such as the dorsal tegmental and posterodorsal tegmental nuclei, which are also implicated in the control of head movements (Wirtshafter and Stratford, 1993).

These results indicate that extinction is the main process targeted by NI-relaxin-3 projections during emotional memory formation. Extinction is context-dependent and other regions including the hippocampus and entorhinal cortex may provide additional regulation of amygdala function, under these conditions. Notably, extinction is dependent on contextual processing, which in turn, is the result of entorhinal-hippocampal interconnections. The hippocampus plays a relevant role in context-dependent extinction (Lissek et al., 2013). The effect of context on extinction becomes clear in the renewal process, which consists of the recall of a cued fear memory that has already been extinguished. Extinction must be done in a different context than acquisition and recall to be effective (Bouton and Ricker, 1994). Notably, transient inactivation of the ventral hippocampus, rich in relaxin-3 fibers, during extinction impaired extinction, as reflected in next-day recall trials (Sierra-Mercado et al., 2011). There is a need to codify the context features during extinction learning, in a process in which the hippocampus plays a central role (Lissek et al., 2013). Thus, the NI projection to the hippocampus may filter relevant or irrelevant contextual features (Szönyi et al., 2019).

The medial and extended amygdala MexA are involved in the control of social behavior (Olucha-Bordonau et al., 2014), and neurons in these areas receive projections from the main and accessory olfactory systems that are associated with the recognition of conspecifics (Scalia and Winans, 1975; Pro-Sistiaga et al., 2007; Gutiérrez-Castellanos et al., 2014). Thus, the MexA is a key element in processing social recognition. In this sense, lesion of the MexA impairs social recognition (Wang et al., 2014). Also, MeA infusion of oxytocin can rescue the impaired social recognition of oxytocin knockout mice (Winslow et al., 2000). Moreover, activation of estrogen receptors in the MexA enhances social recognition (Lymer et al., 2018). Thus, the NI/relaxin-3 projections to these areas may affect social recognition.

The ability of the relaxin-3/RXFP3 system to interfere with social recognition and the possible involvement of the amygdala was assessed in the 3-chamber maze paradigm, which involved two trials (Albert-Gascó et al., 2017). In the first trial—the social test—the test rat was allowed to move between chambers containing either an inanimate object (a toy) or a conspecific. In the second trial—the recognition test—the object was replaced by a novel conspecific and the test rat could move between the familiar and the novel conspecific chambers. Normally, in the social test the rat prefers to explore the conspecific, while during the recognition test, the test rat explores the novel more than the familiar conspecific (Gheusi et al., 1994).

Intracerebroventricular infusion of an RXFP3 agonist, either before the social test or the preference test impaired the capacity to discriminate between novel and familiar conspecific, while the capacity to discriminate between the object and the conspecific subject remained intact (Albert-Gascó et al., 2017). In the same paradigm, excitotoxic lesions of the NI disrupted the recognition test, but did not alter performance in the social test (García-Díaz et al., 2019).

These data suggest a role for relaxin-3/RXFP3 signaling in the control of social recognition and discrimination and related behaviors. The data obtained are compatible with an inhibitory effect of RXFP3 activation on social recognition and indicate that a reduction in NI activity also reduces an inhibitory input to the amygdala centers involved in social recognition. However, at this stage, the specific local circuits and neurons involved in this effect have not been identified. Future research on specific interneuronal targets of NI neurons may elucidate the specific function of these cells in filtering socially relevant information. In such research the use of transgenic rodents in which a particular cell type can be switched on or off in a particular situation may provide additional information, as has been shown for the hippocampus in context conditioning (Szönyi et al., 2019), albeit with some caveats. The relative proportion of RXFP3-expressing neurons in relation to other markers may provide additional cues, as seen for the expression of RXFP3 in relation to oxytocin in the MeA (Albert-Gasco et al., 2019). Notably, the medial and extended amygdala express a high density of RXFP3 (Ma et al., 2007; Smith et al., 2010). Around 60% of medial and extended amygdala neurons co-express RXFP3 and oxytocin receptors (Albert-Gasco et al., 2019), which raises the possibility of an interaction between these signaling systems during social encounters. Recent studies have demonstrated the ability of RXFP3 activation to inhibit the activity of oxytocin neurons in the hypothalamic paraventricular nucleus (Kania et al., 2017, 2020), but putative interactions of relaxin-3 and oxytocin signaling within the amygdala have not been investigated at the behavioral, neurophysiological or cell signaling level.

The restriction of relaxin-3 fibers to the extended amygdala and data obtained from pharmacological and broad lesion approaches indicate an effect on social behavior. However, these approaches lack sufficient accuracy in the present context in which different neuronal types are located in such a small pontine center. The fact that the NI projects at the same time to interrelated emotional and memory processing centers like the hippocampus, prefrontal cortex, and MexA raises the possibility of coordinated control of particular aspects of their function. For example, CRF activation of NI neurons can suppress prefrontal cortex LTP obtained from hippocampal stimulation (Farooq et al., 2013). In the future, it will be necessary to use specific biotechnology tools to determine the exact role of each signaling system in each aspect of social memory and/or interaction.

## Conclusions

There is now considerable anatomical, physiological, molecular, and behavioral evidence supporting a role for the NI in processes associated with memory acquisition, consolidation, retrieval, and/or extinction. NI projections target key structures involved in explicit and implicit memory, including the hippocampus, MS, amygdala, and prefrontal cortex. Characterization of the relative contribution of each NI neuron population, including those containing one or more neuropeptides to each of these targets may shed light on the specific modulation by the NI of the function performed by each of these structures and associated circuits. For example, the NI projection to the septohippocampal system is strongly implicated as a key component of the subcortical system driving hippocampal theta rhythm. More specific and powerful methods based on viral tools containing specific peptide promotors may provide additional insights through the use of optogenetics and chemogenetics in controlled behavioral paradigms (Wykes et al., 2020).

At a neurophysiological level, there is a clear association between NI activity and hippocampal theta rhythm and synchronization between the hippocampus and the NI, but under certain conditions, increased NI activity may focally induce a decrease in hippocampal theta activity *via* inhibition of hippocampal somatostatin interneurons. These data might be best explained based on a role in increasing the “signal-to-noise” in memory processing. Currently, there is a lack of data in other mammalian taxa about the relative proportional occurrence of relaxin-3 neurons in the four centers containing these neurons in rodents (but see Ma et al., 2009b), and further comparative studies will be important for revealing similar or differential functions of this system in particular taxa, including humans, under both normal and pathophysiological conditions.

At a molecular level, RXFP3 activation by relaxin-3 may induce neuronal responses *via* two main signaling pathways, one directly through inhibition of cAMP synthesis that may reduce the level of cAMP-PKA pathway activity, and the other indirectly through altered levels of ERK phosphorylation. The balance between these two pathways and their effects on neuronal ion channels and firing may result in the erasing and enhancing of different aspects of memory formation.

Finally, at a behavioral level, it has become clear that the NI and the relaxin-3/RXFP3 system can modulate declarative memories, as reflected by effects on multiple aspects of spatial processing in the T-maze test and the MWM. Furthermore, NI and relaxin-3/RXFP3 signaling can also influence social recognition and the extinction of fear conditioning. Thus, specific aspects of cognitive and emotional processing can be modulated by particular components of the projections arising from the NI and its associated peptidergic systems.

## Author Contributions

IG-M and FO-B: conceptualization. IG-M, FR-B, AM-O, and FO-B: writing—original draft preparation. HA-G, EC-G, FR-B, AG, and FO-B: writing—review and editing. IG-M and AM-O: figure preparation. All authors have read and agreed to the published version of the manuscript. All authors contributed to the article and approved the submitted version.

## Conflict of Interest

The authors declare that the research was conducted in the absence of any commercial or financial relationships that could be construed as a potential conflict of interest.
